# Adaptive emergency response and dynamic crowd navigation for mobile robot using deep reinforcement learning

**DOI:** 10.3389/frobt.2025.1612392

**Published:** 2025-10-10

**Authors:** Anusha Alexander, V. N. Suchir Vangaveeti, Kalaichelvi Venkatesan, Jinane Mounsef, Karthikeyan Ramanujam

**Affiliations:** 1 Department of Electrical and Electronics Engineering, Birla Institute of Technology and Science, Pilani, Dubai Campus, Dubai International Academic City, Dubai, United Arab Emirates; 2 Department of Computer Science, Birla Institute of Technology and Science, Pilani, Dubai Campus, Dubai International Academic City, Dubai, United Arab Emirates; 3 Electrical Engineering Department, Rochester Institute of Technology, Dubai, United Arab Emirates; 4 Department of Mechanical Engineering, Birla Institute of Technology and Science, Pilani, Dubai Campus, Dubai International Academic City, Dubai, United Arab Emirates

**Keywords:** deep reinforcement learning, mobile robot, deep Q-network, deep deterministic policy gradient, twin delayed DeepDeterministic policy gradient, crowd navigation

## Abstract

Mobile robots have emerged as a reliable solution for dynamic navigation in real-world applications. Effective deployment in high-density crowds and emergency scenarios requires not only accurate path planning but also rapid adaptation to changing environments. However, autonomous navigation in such environments remains a significant challenge, particularly in time-sensitive applications such as emergency response. Existing path planning and reinforcement learning approaches often lack adaptability to uncertainties and time-varying obstacles, thereby making them less suitable for unstructured real-world scenarios. To address these limitations, a Deep Reinforcement Learning (DRL) framework for dynamic crowd navigation using three algorithms, Deep Deterministic Policy Gradient (DDPG), Twin Delayed Deep Deterministic Policy Gradient (TD3), and Deep Q-Network (DQN), is proposed. A context-aware state representation that combines Light Detection and Ranging (LiDAR)-based obstacle perception, goal orientation, and robot kinematics to enhance situational awareness is developed. The proposed framework is implemented in a ROS2 Gazebo simulation environment using the TurtleBot3 platform and tested in challenging scenarios to identify the most effective algorithm. Extensive simulation analysis demonstrates that TD3 outperforms the other approaches in terms of success rate, path efficiency, and collision avoidance. This study contributes a reproducible, constraint-aware DRL navigation architecture suitable for real-time, emergency-oriented mobile robot applications.

## 1 Introduction

Mobile robots navigating in dynamic environments often face challenges such as obstacle prediction and detection, environmental awareness, and adaptive path planning. Real-time adaptation of mobile robots in complex scenarios forms the basis of almost every research and industrial application. The cumulative integration of mobile robots in urban environments, especially for applications in crowd management and emergency response, has always stood to be a significant challenge. In such high-risk environments, the robust-decision making depending on the uncertain conditions and the ability of the mobile robot to manoeuvre quickly and safely is always paramount. This makes robotics a very vital tool in augmenting human efforts to increase the overall safety (Hewawasam et al., 2022). The rapidly increasing demand for autonomous robots in disaster management, with the critical research focus on advanced navigation techniques suiting various uncertain scenarios, is evident. Sensor integration enables a mobile robot to perceive its exposed surroundings with better accuracy, enabling it to detect hazards, avoid obstacles, and make decisions in real time.

Over the last decades, the integration and deployment of mobile robots in emergency response and dynamic crowd navigation have gained significant attention. This is mainly due to the need for rapid and autonomous decision-making in complex urban environments. Traditional navigation and path planning methods often struggle to handle these uncertain environments, particularly in the mentioned complex situations and crowded public spaces like in a mall or public gathering. For a mobile robot to improve response times and ensure safe navigation, it must be able to adapt to uncertain environments involving human movement patterns, shifting impediments, and environmental risks.

Traditional navigation systems that currently exist struggle to adapt to these complex environments. This is mainly due to certain factors such as unexpected obstacles, crowd density, and human unpredictability, as these pose significant risks to both robots and individuals in the vicinity (Zhang et al., 2023). Dynamic path planning algorithms, in contrast to static path planning, adjust routes in response to evolving environmental conditions. They will continuously update the robot’s trajectory based on real-time sensor data. This adaptability is very crucial in dynamic scenarios wherein there is a high chance of an obstacle appearing unexpectedly. In recent years, DRL has emerged as a promising technique for developing adaptive navigation strategies that allow robots to learn optimal behaviours through interaction with their environment (Li P. et al., 2024). DRL algorithms enable robots to dynamically adjust their path planning in dynamic environments, learning to balance competing objectives such as avoiding collisions, minimizing travel time, and ensuring human safety (Ou et al., 2024).

This paper explores the application of DRL in the context of adaptive emergency response and dynamic crowd navigation. Mobile robots learn to navigate these environments, making decisions that ensure safe and efficient movement using DRL, even in rapidly changing scenarios. After doing an intensive literature survey on existing dynamic path planning algorithms, this study aims to identify key advancements and challenges in modern navigation algorithms, mainly DRL techniques. The main objective of this research is to develop an integrated approach that will enable mobile robots to maneuver in a dynamic environment safely and efficiently using the best-performing algorithm. To achieve this, a framework that enables mobile robots to quickly adapt to such situations where crowd density and movement patterns shift unexpectedly, requiring instant decision-making ability using various DRL path planning algorithms is developed. Each algorithm is trained and tested on the same simulation platform, and results are extracted. This research will contribute to the development of an adaptable robotic system for disaster response, reducing the risk to human life and thereby increasing the effectiveness of rescue operations.

While recent works have explored DRL-based navigation strategies, the focus has mostly been on structured or static environments and tends to omit factors critical to real-time navigation in dense dynamic settings. For instance, Bao et al. (2024), proposed a digital twin-assisted DRL planner for multiple AGVs in structured industrial setups, while Liu (2024a) benchmarked DRL algorithms for generic continuous control with a focus on navigation in unpredictable environments. Yu et al. (2023) utilized a vision-based DRL for obstacle avoidance but did not incorporate emergency responsiveness.

In contrast, our work addresses the above limitations by developing a comprehensive DRL framework specially tailored to emergency and crowd-aware navigation. The major contributions of this work are as follows:

• A context-aware DRL-based navigation framework is developed that can enable a real-time mobile robot to adapt to emergency and high-density crowd scenarios.

• A compact and dynamic state representation incorporating LiDAR scan data, robot kinematics, and environmental awareness is designed in a simulation environment.

• Comparison and benchmark evaluation of three DRL algorithms using consistent metrics on the same simulation environment.

• A detailed performance analysis based on collision rate, success rate, path efficiency, and computational complexity is provided.

• A robust reward-shaping mechanism suited for both emergency and crowded scenarios, and its impact on policy learning is analyzed.

• A unique and scalable simulation setup based on ROS2, Gazebo, and real-world kinematic constraints is implemented, making the framework easily extensible to hybrid DRL approaches.

This paper is divided into the following sections. Section 2 will explain the existing literature surveys of path planning algorithms, also diving deeply into DRL techniques and also highlights the gaps in existing literature. Section 3 explains the framework of path planning algorithms and related DRL algorithms. The simulation results and discussions are elaborated in Section 4. Finally, the experimental results are tabulated, and the best algorithm for adaptive response and crowd management implementation is identified. The later section explains how efficiently this implementation can be done in real time by incorporating the analysis of this study.

## 2 Related works

### 2.1 Dynamic path planning

Path planning remains the core challenge in robotics, mainly while navigating in diverse environments, which include both static and dynamic environments. In (Liu and Zhang, 2022), a novel methodology is presented based on dynamic programming to generate optimal paths for mobile robots employing Model Predictive Control (MPC) for path tracking. It is shown that the proposed approach addresses environmental complexities and the robot navigates through different terrains. Compared to Artificial Potential Fields (APF) and PSO combined techniques, this highlights a notable improvement in path smoothness and accuracy with low computational demands. However, while simulations are promising, the method’s performance in an environment with high sensory noise is to be studied in detail.

Reinforcement learning techniques have become increasingly popular due to their adaptability in dynamic environments. Xiao et al. (2024) have proposed a Q-learning-based trajectory tracking approach. It integrates real-time obstacle avoidance capabilities and has optimized efficiency in the decision-making process. While simulation results validate the method’s effectiveness, real world deployment remains a challenge, especially in complex environments. To address the main challenges in achieving high success rates and efficient training (Ou et al., 2024), DRL approaches. By leveraging pre-trained models from static environments, the proposed algorithm adapts to quick dynamic changes. However, adaptation to real-world applications is challenging when exposed to complex environments and sensor noise, which necessitates future work on optimizing training speeds, sample efficiency, and adaptation capabilities. Additionally, combining DRL with classical path planning can yield better global planning and local obstacle avoidance.

Recent research by Zhao et al. (2023) has leveraged a dynamic path planning algorithm based on Gaussian probability models. This approach will combine global reference path optimization using quadratic programming with local planning. Simulation results in ROS/Rviz show significant efficiency improvements compared to traditional RRT algorithms. Additionally, field tests confirmed the algorithm’s practicality in autonomous and effective obstacle avoidance. Traditional A* algorithms often struggle with difficulties in handling unknown obstacles. ASL-DWA is used in (Liu and Zhang, 2022), which enhances the A* algorithm by incorporating a heuristic function that can combine Euclidean distance with point-to-line distance, thereby decreasing the number of search nodes. Experimental comparisons in three distinct indoor environments highlight its better significance over traditional methods.

The traditional Harmony Search (HS) algorithm has proved its effectiveness in many optimization problems; however, it requires enhancements to effectively handle the dynamic world. Quan et al. (2021) introduced an improved self-adaptive HS algorithm combined with the Morphin algorithm for path planning. Relative simulations demonstrate better convergence and accuracy over Particle Swarm Optimization and Whale Optimization Algorithm. Future research can include other hybrid strategies within the HS framework. The Dynamic Window Approach (DWA) is another method used for path planning; however, a primary limitation is that it is dependent on the accurate tuning of the objective function’s weight parameter.

Abubakr et al. (2022) proposed an Adaptive Dynamic Window Approach (ADWA), which employs a fuzzy logic controller to dynamically adjust the weight parameters based on real-time data. The comparative analysis demonstrates superior performance and shows improved path length. However, further refining of weight and exposure to diverse environments needs to be included to understand the efficiency of the algorithm in complex scenarios. The recent advancements focus on integrating machine learning techniques such as reinforcement learning (Zhang et al., 2023) and deep learning techniques. Various strategies have been explored to improve efficiency and adaptability in unknown environments in the field of multi-swarm robotic systems. It is seen that initial research mainly relied on either static or reactive approaches, where robots follow a predefined path. One major remarkable advancement is the use of polynomial fitting for predictions of trajectories, as seen in (Song et al., 2024), where algorithms dynamically update the positions of targets based on sampled trajectory points. It is also seen that when combined with reinforcement learning, the system performs better, enhancing path planning and target prediction. DRL has further enhanced the system by allowing robots to learn from their immediate environment and thereby improving their performance.

### 2.2 Deep reinforcement learning (DRL)

The article (Kargin and Kołota, 2023) applied the DDPG algorithm to address the complex control challenges of continuum robots that belong to a class of robots that are highly flexible and are characterized by a continuous structure, allowing high dexterity in constrained or complex environments. However, the control of these robots modulates unique challenges. This is mainly due to the continuous nature of their movements and highly nonlinear kinematics, which contrast with the traditional robotic systems with discrete joints. In (Lee et al., 2024), the authors explored the integration of Digital Twin technology with DDPG to enhance communication efficiency in UAV networks. It demonstrated that the approach maximizes sum-rate performance effectively, highlighting the potential of the algorithm’s application in complex communication scenarios. In (Liu et al., 2024), Liu et al. introduced a learning method, applying DDPG to navigate dynamic environments. The study illustrates TD3’s proficiency in autonomous control by addressing issues such as dynamic path adjustment and obstacle avoidance. DDPG’s capability in dynamic robotic control is shown in the study, and the challenges, such as obstacle avoidance and dynamic path navigation, are addressed.

The authors of (Jeng and Chiang, 2023) compare TD3 and DDPG in tasks such as autonomous navigation. The study demonstrates that employing a survival penalty function to address limited reward problems reveals TD3’s superiority over DDPG in dynamic environments regarding stability and convergence rate. It emphasizes the extent to which DQN, DDPG, and TD3 algorithms enhance robotic capabilities, particularly in the context of dynamic path planning, when considered in their entirety. In (Antonyshyn and Givigi, 2024), Antonyshyn and Givigi introduced a system that employs DQN in scenarios with both sparse and dense rewards. The research demonstrates how DQN enhances predictive control in robotic systems by adeptly achieving a balance between exploration and exploitation. A new DDPG algorithm with an improved experience replay mechanism and mixed rewards is proposed in (Dong et al., 2023). This approach improves robotic limb control by efficiently managing sluggish convergence and local optima. The authors investigated the utilization of TD3 in the training of Spiking Neural Networks (SNNs) for robotic control (Akl et al., 2023). The study has demonstrated that the combination of TD3 and SNN results in scalable and robust learning, which presents intriguing opportunities for neuromorphic engineering in robotics.

It is understood that recent studies continue to emphasize hybrid and adaptive approaches to improve performance in unstructured environments. As mentioned before (Bao et al., 2024), highlights the potential of simulation-to-reality alignment through virtual environments. While effective in industrial layouts, the approach has limited scalability to dense crowds. As referred initially, Yu et al. (2023) incorporated vision-based perception with DRL for obstacle avoidance but showed a lack of responsiveness in unpredictable emergency settings. In a similar context, Wang et al. (2024) introduced a memory-augmented actor-critic architecture to improve long-term planning in robots; however, high computational complexity hinders real-time deployment. A novel safe exploration technique was introduced by Zhang et al. (2025), applying curriculum learning in DRL, which gradually exposes agents to complex environments, improving stability but requiring extensive pre-training phases.

In terms of algorithm benchmarking, the authors (Liu, 2024b) conducted a diligent comparison of DDPG, TD3, Soft Actor Critic (SAC), and Proximal Policy Optimization (PPO) in continuous control domains but lacked crowd-focused evaluation. Antonyshyn and Givigi (Antonyshyn and Givigi, 2024) analyzed DQN under varying reward sparsity conditions and demonstrated its suitability for sparse emergency signals. Meanwhile, a study by Kim et al. (2025) applied multi-agent TD3 in cooperative rescue scenarios, achieving high coordination success but depending heavily on predefined communication protocols. These works indicate a growing interest in robustness, safety, and contextual decision-making; however, very few have explicitly addressed DRL-driven emergency response in dynamic human environments. The present study fills this gap by not only comparing TD3, DDPG, and DQN but also its application in emergency crowd navigation. It also emphasize on contextual perception, reward shaping, and safety constraints within a simulation framework thereby making this study unique and building a framework easily extensible to hybrid DRL approaches.

Mobile robot navigation has demonstrated its strength in various kinds of surroundings by employing a wide range of DRL algorithms. Among these, it is seen that DQN, DDPG, and TD3 are emerging as prominent approaches, mainly due to their adaptability. From the above literature works it’s evident that DQN is effective in discrete actions and constrained spaces, and the other two algorithms show superior performance in real-time navigation tasks. Despite the evolving body of research around these three algorithms, there remains a gap of comparative evaluations focusing on emergency response and dynamic crowd navigation, where the performance benchmarks of adaptability, responsiveness, and collision avoidance are critical. As such, this research will aim to conduct a focused study on the three state-of-the art DRL algorithms, evaluating their performance in unpredictable and highly dynamic environments. By analyzing their decision-making efficiency, trajectory smoothness, and real-time adaptability, we will be able to determine the most suitable algorithm for mobile robot application in emergency and crowd-dense scenarios.

## 3 Static and dynamic path planning

Path planning for mobile robots has evolved significantly over time, from rigid, preset routes to highly adaptable and dynamic navigation methods. Robots initially followed static routes, which were preset trajectories or fixed waypoints, to function in controlled environments like warehouses. The shortest path through a mapped environment was the primary objective of traditional techniques that used grid-based or graph-based algorithms, such as the A* and Dijkstra algorithms. Static path planning decreased overall operational efficiency in dynamic environments because it was inadequate to handle unforeseen obstacles and changes in the real-time environment, even though it was useful in simple, controlled situations. These static path-planning methods work best in environments that were actually controlled, predictable, and had obstacles and operating conditions that were mostly constant.

Using a pre-existing map or environment model, static path planning methods predict a robot’s future route. This approach is commonly seen in warehouse automation, where robots are programmed to follow floor markings, magnetic strips, or predefined coordinates. Algorithms such as Dijkstra’s and A* are frequently used to calculate these paths and have thus gained widespread popularity. Due to its predictability, repeatability, and ease of use, static path planning is well-suited for structured, unchanging environments. However, it struggles with unforeseen obstacles or human interference. Delays and poor adaptability typically result from the need for manual intervention, recalibration, or environmental changes.

Contrary to the above, a robot’s dynamic path planning enables real-time navigation and travel route modification based on environmental changes. It incorporates sensor fusion techniques, machine learning, and DRL to help robots to recognize and respond to a shift in their surroundings. Dynamic algorithms such as Rapidly-exploring Random Tree (RRT) and Dynamic Window Approach (DWA) allow a robot to independently identify obstacles and human activity and adjust its path accordingly. In environments with frequent changes mainly due to human presence, shifting inventory, or other moving automation, dynamic path planning is essential. This ability enhances efficiency, reduces the risk of collisions, and builds resilience within the system. While static planning is beneficial, it is limited to simplistic and constant environments where replication is vital. However, dynamic planning is best suited for intricate and disorderly environments without certainty of factors and conditions. The responsiveness and efficiency of dynamic path planning are superior to those of static systems in terms of both of these characteristics. In order to accomplish the goal, it is necessary to combine sensor data in real time, to make intelligent decisions, and to use learning models that are flexible. As warehouse and industrial environments become more complex, the demand for dynamic path planning continues to grow. The integration of AI and robotics not only simplifies human-robot collaboration but also fosters greater autonomy (Li Keqin et al., 2024; Bao et al., 2024; Chen et al., 2022).

### 3.1 Framework of dynamic path planning using DRL algorithms

**FIGURE 1:**
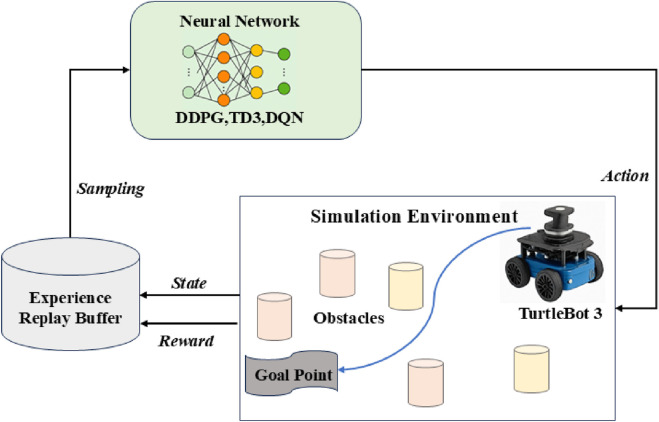
Framework of the dynamic path planning architecture based on DRL algorithm.

Dynamic path planning in real-world settings calls for robots to make smart navigation choices in the presence of all impediments, circumstances, and time-sensitive activities. This system uses DRL algorithms, allowing mobile robots to acquire adaptive navigation techniques by interacting with their environment to meet these difficulties. The system lets agents constantly assess their status, select the best behaviors, and get feedback in the form of incentives by use of a Markov Decision Process (MDP) modeling of the environment. To guarantee that robots can safely and effectively reach their objective sites in complicated, dynamic situations like emergency response or crowd navigation, the suggested method combines sensor data, reward shaping, and decision-making modules (Chen et al., 2023). Figure 1 is the framework of the Dynamic Path Planning Architecture. It shows the end-to-end system integrating state input, DRL agent, and actuator output for robot navigation.

#### 3.1.1 State space and action space

The mobile robot uses a number of sensory inputs that perceive the environment. A 360° scan with 180 data points is provided by the LiDAR per frame, thereby ensuring complete obstacle detection. Additionally, the mobile robot processes the relative position of the target, the distance to the nearest obstacles, and its own current velocity and angular acceleration, enabling situational awareness. The constructed context-aware state vector integrates both spatial and temporal perception.

Depending on the type of reinforcement learning, action space varies (Xue et al., 2024). The 360-degree LiDAR captures both static and dynamic obstacles in real time. As moving agents or obstacles enter or exit the path of the mobile robot, the laser scan values update the state representation, enabling the agent to adapt its policy accordingly. This will allow the system to navigate along dynamically changing environments while perceiving time-varying obstacles. However, static structure remains consistent in the state, while moving obstacles alter the state vector across time steps, reinforcing the temporal aspect of learning. For DDPG and TD3, the mobile robot commutes within a continuous action space, and it controls its linear velocity v∈[0,0.22] m/s and angular velocity w∈[0,0.22] rad/s, allowing for smooth and precise movement. In contrast, DQN employs a discrete action space, restricting the robot to predefined movement commands such as “Move Forward,” “Turn Left,” and “Turn Right,” simplifying decision-making but limiting flexibility in complex navigation scenarios. This modular state-action modeling is essential to enable both fine-grained control for DDPG and TD3 and efficient decision-making in constrained environments for DQN.

The path planning problem for a mobile robot navigating dynamic environments can be formulated as a Markov Decision Process (MDP) described by the tuple 〈 S, A, P, R, γ〉 . Here S represents the state space, encoding the robot’s perception and internal dynamics, and A denotes the action space, comprising motion commands. $P\left(s^{\prime }|s,a\right)$ is the transition probability function. R(s,a) is the reward function that guides learning. γ∈[0,1] is the discount factor for future rewards. In our framework, the state $s_{t}\in S$ encapsulates the robot’s sensory input and trajectory context (Chen et al., 2023). It is defined as:

$$s_{t}=\left[{\text{scan}}_{t},v_{t-1},w_{t-1},\theta_{\text{diff}},d_{t}\right]$$ (1)

where ${scan}_{t}$ is defined as the laser scan data at time t, $v_{t-1}$ is the linear velocity of the turtlebot at the previous time step t-1, $w_{t-1}$ is the angular velocity at time t-1, $\theta_{\text{diff}}$ is the heading angle difference between the robot’s current orientation and the direction toward the goal. The distance to the goal is computed as:

$$d_{\text{t}}=\sqrt{{\left(x_{\text{goal}}-x_{\text{t}}\right)}^{2}+{\left(y_{\text{goal}}-yt\right)}^{2}}$$ (2)

The heading angle difference is given by:

$$\theta_{\text{goal}}=arctan\;2\left(y_{\text{goal}}-y_{\text{t}},x_{\text{goal}}-x_{\text{t}}\right)$$ (3)

These Equations 1–3, define the expected return and update rules central to value-based and policy gradient methods.

$$\theta_{\text{diff}}=\theta_{\text{goal}}-\theta_{\text{t}}$$ (4)

The robot’s orientation t is derived from odometry by converting quaternion values into Euler angles:

$$\theta_{\text{t}}=euler\;from\;quaternion\left(q\right)$$ (5)

The action $a_{t}\in A$ comprises continuous control commands, defined as:

$$a_{t}=\left[v_{t},\omega_{t}\right],\;\text{where }\;v_{t}\in \left[0,0.22\right]\text{ m/s},\;\omega_{t}\in \left[-2,2\right]\text{ rad/s}$$ (6)

This continuous control allows more precise maneuvering than discretized actions, which is critical in cluttered and dynamic environments such as emergency scenarios or crowded spaces. In reinforcement learning, the agent’s objective is to maximize the expected cumulative discounted reward:

$$J\left(\pi \right)=E_{\pi }\left[\overset{\infty }{\underset{t=0}{\sum }}\gamma^{t}R\left(s_{t},a_{t}\right)\right]$$ (7)

Here J(π) is defined as the performance objective of a policy π , $E_{\pi }$ is the expected value when the agent follows a policy. π . $\left[\sum_{t=0}^{\infty }\gamma^{t}R\left(s_{t},a_{t}\right)\right]$ is the cumulative discounted reward. This is solved using the Bellman Optimality Equation, which defines the optimal value function V*(s) and Q*(s,a) the optimal action-value function as:

$$V^{*}\;\left(s\right)=\underset{a}{\max}\left[R\left(s,a\right)+\gamma \underset{s^{\prime }}{\sum }P\left(s^{\prime }|s,a\right)V^{*}\;\left(s^{\prime }\right)\right]$$ (8)

Alternatively, the optimal action-value function Q*(s,a) is given by:

$$Q^{*}\;\left(s,a\right)=R\left(s,a\right)+\gamma \;E_{s^{\prime }}\left[\underset{a^{\prime }}{\max}Q^{*}\;\left(s^{\prime },a^{\prime }\right)\right]$$ (9)

Here R(s,a) is the immediate reward for taking action a in the state s , γ which is the discount factor ∈ [0,1). The transition probability of reaching the next state $s^{\prime }$ wrt to the current state and action is denoted as $P\left(s^{\prime }|s,a\right)$ . Expectation over possible next states $s^{\prime }$ is $E_{s^{\prime }}$ and $\max_{a\prime }Q^{*}\left(s^{\prime },a^{\prime }\right)$ indicates the agent will choose the best possible action in the future. The above-defined equations act as the basic theoretical foundation of Q-learning and value iteration in dynamic programming. Equations 4–9 derive the actor-critic gradient used in DDPG and TD3 to update their policies with respect to continuous actions.

Using neural networks, deep reinforcement learning algorithms like DDPG and TD3 mimic these functions, hence allowing policy learning in high-dimensional and continuous action spaces. Our approach guarantees real-time responsiveness and adaptation to complicated navigation issues by using a smooth continuous control space and an informative, compact state representation. In a simulated environment, Bellman-based optimization enables the agent to acquire optimal long-term navigation techniques. It is done through trial and error, whereby transferring them to actual robotic systems (Lillicrap, 2015; Duan, 2016).

#### 3.1.2 Reward function design

The reward function is designed to encourage efficient and safe navigation while penalizing undesired behaviors. It consists of multiple components that guide the learning process by reinforcing positive actions and discouraging negative ones. The function (Equation 10) begins by calculating several individual reward components based on the robot’s orientation, movement actions, proximity to the goal, and obstacle avoidance. For example, $r_{\text{yaw}}$ is calculated as the negative absolute value of the goal angle (θ) , which means that any deviation from the desired orientation results in a penalty. This encourages the robot to maintain a trajectory aligned with the target direction. Similarly, $r_{\text{vangular}}$ penalizes large angular velocities by subtracting the square of the angular action, thus discouraging erratic rotational movements that could destabilize the robot’s path.

$$r_{t}=r_{\text{yaw}}+r_{\text{distance}}+r_{\text{obstacle}}+r_{\text{vlinear}}+r_{\text{vangular}}-r_{\text{offset}}+r_{\text{terminal}}$$ (10)

where:

• $r_{\text{yaw}}=-|\theta_{\text{goal}}|$ : *Penalizes deviation from the goal direction*

• $r_{\text{distance}}=\frac{2\cdot d_{\text{initial}}}{d_{\text{initial}}+d_{\text{goal}}}-1$ : *Encourages goal proximity*

• $r_{\text{obstacle}}=-20$ : *Penalty when closer than* 0.22 m *to any obstacle*

• $r_{\text{vlinear}}=-{\left(\left(v_{\text{max}}-v_{\text{action}}\right)\cdot k\right)}^{2}$ : *Penalizes erratic linear speeds*

• $r_{\text{vangular}}=-\omega^{2}$ : *Discourages large angular velocities*

• $r_{\text{offset}}=1$ : *Baseline shift*

• $r_{\text{terminal}}$ : *+2500 if goal is reached; -2000 if collision occurs*

These components will collectively structure the learning signal to ensure a tradeoff between path efficiency, safety, and goal attainment, thereby shaping both the value function and the control policy. The reward values are scaled to balance short-term responsiveness with long-term goal achievement. A pseudo-algorithm (Algorithm 1) shows that these elements taken together guarantee that the robot not only concentrates on promptly attaining the goal but also on preserving an efficient and secure path. This reward function plays a central role in shaping the learned behavior of the agent by implicitly influencing the Q-function in DQN and the state value function in DDPG and TD3. The individual components of reward are carefully designed to balance goal seeking, safety, and motion smoothness. For instance, $r_{\text{distance}}$ ensures continuous motivation towards the goal, whereas $r_{\text{obstacle}}$ performs the duty of sharp penalty imposition to ensure collision avoidance. The angular and linear velocity penalties act as regularization terms to promote stable control, and $r_{\text{terminal}}$ calculates rewards based on goal state achievement.

Algorithm 1 Reward Calculation for Mobile Robot.

1: **Input:** Current state $s_{t}$ , action $a_{t}$ , laser scan L , goal position g

2: **Output:** Reward $r_{t}$

3: Compute heading error: $\theta_{\text{goal}}\leftarrow \text{angle\_diff}\left(robot\text{\_}heading,g\right)$

4: Compute distance to goal: $d_{\text{goal}}\leftarrow \|robot\text{\_}position-g\|$

5: Normalize distance: $r_{\text{distance}}\leftarrow \frac{2\cdot d_{\text{initial}}}{d_{\text{initial}}+d_{\text{goal}}}-1$

6: Compute obstacle penalty: $r_{\text{obstacle}}\leftarrow -20$ if min(L)<0.22 m else 0
7: Compute yaw alignment penalty: $r_{\text{yaw}}\leftarrow -|\theta_{\text{goal}}|$

8: Compute velocity penalties:
9: $r_{\text{vlinear}}\leftarrow -{\left(\left(v_{\text{max}}-v_{\mathrm{action}}\right)\cdot k\right)}^{2}$

10: $r_{\text{vangular}}\leftarrow -\omega^{2}$

11: Set baseline offset: $r_{\text{offset}}\leftarrow 1$

12: Compute terminal reward:
13: **if** goal_reached **then**
14: $r_{\text{terminal}}\leftarrow +2500$

15: **else if** collision **then**
16: $r_{\text{terminal}}\leftarrow -2000$

17: **else**
18: $r_{\text{terminal}}\leftarrow 0$

19: **end if**
20: Compute total reward:

$r_{t}\leftarrow r_{\text{yaw}}+r_{\text{distance}}+r_{\text{obstacle}}+r_{\text{vlinear}}$ $\;+\;r_{\text{vangular}}-r_{\text{offset}}+r_{\text{terminal}}$

21: **return** $r_{t}$

This structure ensures that the agent will not only learn to reach the goal position efficiently but also internalize policies that are safe and feasible dynamically. Our reward-shaping strategy is consistent with reinforcement learning theory, where reward design directly determines the optimal learned policy. This also aligns with the findings from Tutsoy and Brown (Tutsoy and Brown, 2015) that demonstrate piecewise-linear control objectives can be embedded into the reward structure to ensure convergence and desirable control behaviors. Our framework leverages this principle by ensuring that positive rewards drive goal-seeking, while heavy obstacle penalties reinforce collision avoidance, resulting in well-shaped policies capable of dynamic crowd navigation.

#### 3.1.3 Learning model and training constraints

The learning model setup involves training DRL agent in Gazebo using ROS2 Foxy and PyTorch. Below are the training parameters and constraints included:

• Training episodes: 8000

• Max episode duration: 50 s

• Time step: 0.01 s

• Maximum linear velocity: 0.22 m/s

• Maximum angular velocity: ± 2 rad/s

• Minimum safe distance from obstacles: 0.22 m

• Discount factor: γ=0.99

• Batch sizes: 128 for DQN, 1024 for DDPG/TD3

These constraints impose safe exploration while enabling learning under physical dynamics. Each algorithm is trained with a prioritized experience replay mechanism and is periodically evaluated to ensure convergence and robustness and to identify the most efficient algorithm. These physical and safety constraints are embedded in the simulation environment, and the DRL agent learns to operate effectively within them. For example, an instance exceeding the safe distance threshold triggers penalties in the reward function, while timeouts prevent inefficient policies. This bounded learning approach will ensure the agent outperforms in not only finding the optimal policies but also does so while respecting the real-world limitations such as speed caps, response deadlines, and collision avoidance.

Although the training evaluations are conducted in a simulation environment, the framework considers multiple real-world constraints, including obstacle proximity, maximum velocity, and time limits. These constraints reflect physical limitations that prevail in the actual robotic platforms. Additionally, the robot operates under nonlinear dynamics, where control inputs such as velocity and rotation interact with environment perception in a strongly coupled manner.

In real-world robotic applications, multiple forms of uncertainty influence navigation safety and performance. Some of these are as listed:

• Internal uncertainties, mainly due to actuator delays, sensor inaccuracies, or localization drift;

• External uncertainties, which include unpredictable crowd movements and various changes in environment;

• Parametric uncertainties such as robot mass, friction, and inertia;

• Non-parametric ones such as human behavior and environmental stochasticity.

While the simulation abstracts away certain disturbances, our model accounts for uncertainty by integrating randomized obstacle motion and dynamic goal placement, thereby training the DRL agent to generalize across variable situations. This is improving the robustness of the learned policy against unexpected conditions.

### 3.2 Path planning algorithm

**FIGURE 2:**
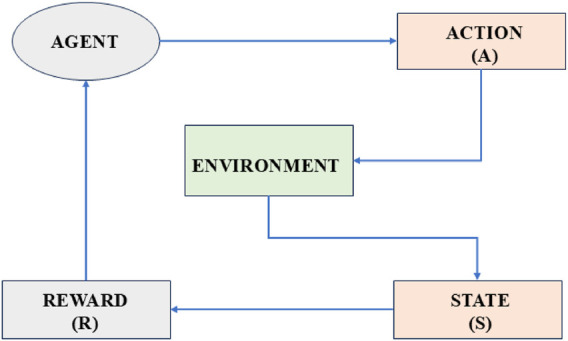
Workflow of the basic reinforcement learning architecture.

DRL has emerged as a powerful tool, allowing robots to learn adaptive navigation strategies through experience. DRL-based methods continuously update their decision-making policies by interacting with the environment, unlike traditional algorithms. DRL improves their ability to handle unforeseen obstacles and changes in crowd behavior through continuous learning. Figure 2 details basic reinforcement learning architecture. It presents the general RL loop structure, depicting environmental interaction, reward feedback, and policy learning. The architecture of reinforcement learning has a few key components, which work together like a feedback loop, such as the agent, which is the decision-maker. This is the algorithm that tries to learn the best actions to take. Environment or world where the agent operates. It responds to the agent’s actions and provides feedback. State (S) is the current situation or condition of the environment, and action (A) is the decision of the agent according to which it acts. The Reward (R) is defined as the feedback from the environment. Positive rewards for good actions are basically like a treat, and negative rewards are penalties. Policy (π) is the strategy the agent follows to decide which action to take based on the current state. The future rewards the agent can expect from a state if it follows the policy are predicted by the value function (V). Q-Function predicts the future rewards for a state-action pair that defines how good it is to take a specific action in a specific state (Chen et al., 2023).

Here in the block diagram the agent takes an action (A) based on the current state (S) using a learned policy (π) . The environment changes based on the action and provides a new state (S) and a reward (R). The agent updates its value function (V) and Q-function (Q) based on the reward and new state. The agent refines its policy (π) to improve future decision-making. This paper focuses on three widely used DRL algorithms, such as DDPG, which is a model-free, off-policy algorithm that enables continuous action control for smooth trajectory planning. TD3 is an improved version of DDPG that reduces overestimation bias and enhances learning stability. DQN can be described as a discrete-action reinforcement learning approach effective for high-dimensional navigation tasks. We will work on all three algorithms in a simulation environment and find out which algorithm maximizes the total reward over time by finding the best policy (Hamid et al., 2024; Kappagantula and Mannayee, 2024).

#### 3.2.1 Deep Q-Network (DQN)

DQN is a value-based DRL algorithm designed for discrete action spaces where the agent selects actions based on predicted Q-values. DQN approximates the Q-value function Q (s,a) using a deep neural network. The Q-value represents the expected future reward of taking action a in state s . It uses the Bellman Equation 11 (Zhang, 2024) to update Q-values as shown below. Here Q(s,a) : the current Q-value of taking action a in state s , representing the expected total return; r : the immediate reward received after taking action a in state s ; γ : the discount factor, 0≤γ≤1 , which determines the importance of future rewards; $s^{\prime }$ : the next state after taking action a from state s ; $a^{\prime }$ : a possible action in the next state $s^{\prime }$ ; $\max_{a^{\prime }}Q\left(s^{\prime },a^{\prime }\right)$ : the maximum expected future reward attainable from the next state $s^{\prime }$ .

$$Q\left(s,a\right)\leftarrow r+\gamma \underset{a^{\prime }}{\max}Q\left(s^{\prime },a^{\prime }\right)$$ (11)

**FIGURE 3:**
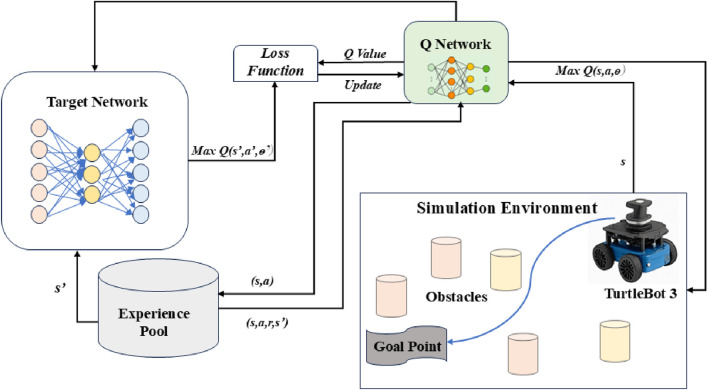
Framework of the DQN algorithm proposed.

In (Zhang, 2024) the authors introduced a Deep Q-Learning framework, which integrates RL with neural networks to enhance accuracy, stability, and scalability in disease prediction using Electronic Health Records. This shows a real-time application demonstrating the applicability of DQN in handling complex networks. It introduces key improvements over vanilla Q-learning in experience replay, where it stores past experiences and samples them randomly to break the correlation between experiences. It also maintains a separate target network to stabilize training by updating it slowly. Its application can be best applied in discrete action spaces such as grid world, Atari games, and so on. Also, its application lies in environments with a small or moderate number of actions. However, the major limitation is that it cannot handle continuous action spaces and struggles with high-dimensional or complex action spaces. Figure 3 describes the framework of the DQN algorithm. The various details of the DQN’s internal components showing Q-network updates and discrete action selection are demonstrated.

#### 3.2.2 Deep Deterministic Policy Gradient (DDPG)

DDPG is a model-free, off-policy RL designed for continuous action spaces and is based on the Deterministic Policy Gradient (DPG) algorithm and DQN. This algorithm uses two neural networks mainly known as the actor network and the critic network. In the actor network, it outputs the best action directly instead of Q-values. And in the critic network, it estimates the Q-value of the state-action pair. The below Equation 12 shows the updates using policy gradients:

$$\nabla_{\theta^{\mu }}J\approx E\left[\nabla_{a}Q\left(s,a|\theta^{Q}\right){\left|\right.}_{a=\mu \left(s\right)}\cdot \nabla_{\theta^{\mu }}\mu \left(s\right)\right]$$ (12)

The term $\nabla_{\theta^{\mu }}J$ represents the gradient of the expected return J with respect to the actor’s parameters $\theta^{\mu }$ . The operator E[⋅] denotes the expectation, typically computed as an average over sampled states from the replay buffer. The function $Q\left(s,a|\theta^{Q}\right)$ is the critic network, which estimates the Q-value of taking action a in state s , parameterized by $\theta^{Q}$ . The expression $\nabla_{a}Q\left(s,a|\theta^{Q}\right){\left|\right.}_{a=\mu \left(s\right)}$ is the gradient of the Q-function with respect to the action, evaluated at the action outputted by the actor. The function μ(s) represents the actor network, which outputs a deterministic action given a state s . Finally, $\nabla_{\theta^{\mu }}\mu \left(s\right)$ is the gradient of the actor’s output with respect to its parameters $\theta^{\mu }$ .

**FIGURE 4:**
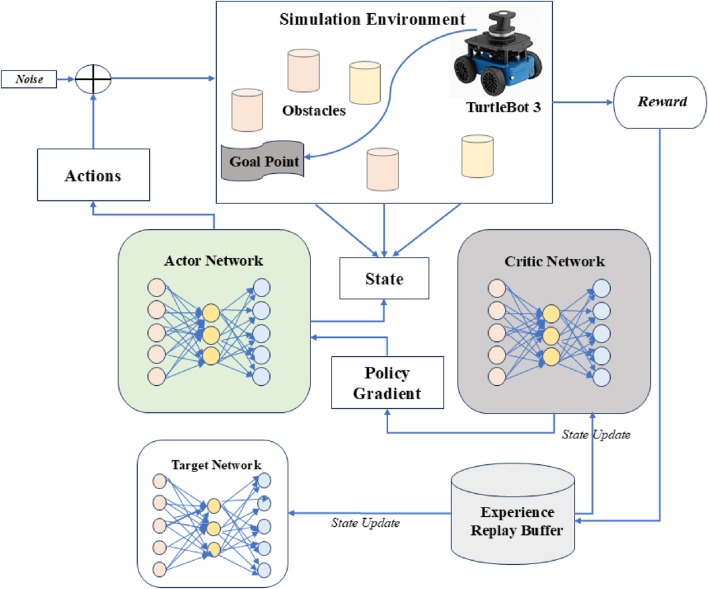
Framework of DDPG algorithm proposed.

Figure 4 describes the framework of the DDPG algorithm. The figure depicts the continuous control learning process with actor-critic networks and target updates. It learns by minimizing the mean squared error between the predicted and target Q-values as shown below Equation 13:

$$L={\left(r+\gamma Q^{\prime }\left(s^{\prime },\mu^{\prime }\left(s^{\prime }\right)\right)-Q\left(s,a\right)\right)}^{2}$$ (13)

The loss function L represents the mean squared error between the predicted Q-value and the target Q-value. The term r is the reward received after taking action a in state s . The discount factor γ determines how much future rewards are considered in the target calculation. The target Q-value is computed using the target critic network $Q^{\prime }\left(s^{\prime },\mu^{\prime }\left(s^{\prime }\right)\right)$ , where $s^{\prime }$ is the next state and $\mu^{\prime }\left(s^{\prime }\right)$ is the action provided by the target actor network for that next state. The main critic network estimates the current Q-value as Q(s,a) . This loss is minimized to train the critic to more accurately estimate the expected return.

It uses experience replay and target networks like DQN and continuous action output with a deterministic policy. It is best suited for continuous action spaces like robot control, stock trading, and tasks that require fine-grained action control. However, the algorithm is prone to overestimation of Q-values, which leads to suboptimal policies, and has high sensitivity to hyperparameters. Recent advancements, such as the ETGL-DDPG algorithm, have been proposed to address these challenges by introducing enhanced exploration strategies and improved experience replay mechanisms (Futuhi, 2024).

#### 3.2.3 Twin Delayed Deep Deterministic Policy Gradient (TD3)

An improved DDPG is TD3, which works on addressing the key weaknesses of DDPG by reducing Q-value overestimation and increasing training stability. It is built on DDPG but introduces three key improvements to improve its efficiency. It is clipped Double Q-Learning and uses two critic networks and takes the minimum Q-value to reduce overestimation as shown below Equation 14:

$$Q\left(s,a\right)=\min\left(Q_{1}\left(s,a\right),\;Q_{2}\left(s,a\right)\right)$$ (14)

**FIGURE 5:**
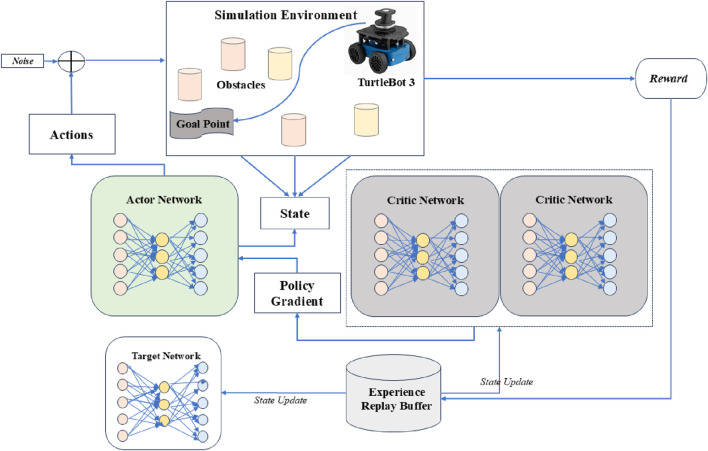
Framework of TD3 algorithm proposed.

The above conservative estimate helps improve stability and robustness during training. Equations 11–14 benchmark the efficiency and safety of learned navigation policies. It also updates the actor network less frequently than the critic to avoid destabilizing learning. Target smoothing is also done automatically by adding small noise to the target action to make the value function smoother and reduce overfitting. Due to its best accuracy, it is used for continuous action spaces and tasks requiring high precision and stability, such as robotic arm control. However, due to dual critic networks, it is computationally more expensive than DDPG and has slower training due to delayed updates (Fujita and Maeda, 2018; Husam, 2024). Figure 5 describes the framework of the TD3 algorithm. It illustrates TD3’s dual critic structure, delayed policy updates, and target smoothing techniques.

## 4 Simulation analysis and discussion

This section describes the simulation environment and the DRL framework used to train the Turtlebot three robot for adaptive emergency response and dynamic crowd navigation.

### 4.1 Simulation platform and environment

The experimental configuration comprises an Ubuntu 20.04 operating system, with PyTorch 1.10.0+cu113 as the deep learning framework, running on an NVIDIA GeForce 4070 GPU (8 GB memory) for accelerated computations. The simulation environment is created using Gazebo 11, while the Turtlebot 3, a tiny differential-drive mobile robot based on ROS Foxy, serves as the core robotic platform. The 360° laser scanner (LiDAR) offers real-time ambient awareness, enabling obstacle recognition and dynamic object tracking. The robot’s control system is based on ROS Foxy, allowing seamless communication between perception, navigation, and control modules for reinforcement learning and adaptive decision-making in challenging scenarios.

**FIGURE 6:**
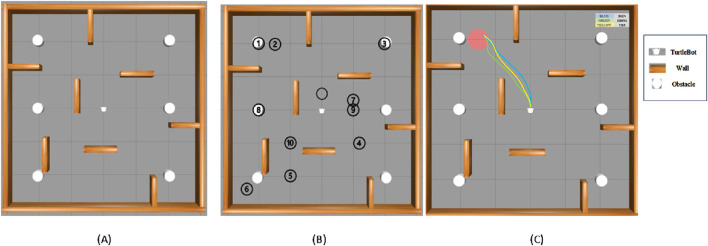
Model showcasing the experimental environment in Gazebo Simulation Space, **(A)** with no goal, **(B)** all 10 goals setup and **(C)** navigation path of three algorithms.

The simulation environment is designed to replicate real-world scenarios where dynamic items move unpredictably, resembling a robot navigating a mall or an emergency evacuation. These devices show incredibly random motion, successfully simulating hectic scenes in malls, warehouses, or disaster scenarios. Additionally, static obstructions such as walls, furniture, and debris add further navigation complexity. Figure 6 displays the gazebo simulation space used for baseline environment testing with the first one with no goals, all 10 goals depicted, and the navigation path of three algorithms, which demonstrates the visual comparison of real-time trajectories generated by all three under identical conditions. The white objects are the randomly moving obstacles, and the brown objects are the static wall separation. The robot’s primary function is to reach preset target locations, such as an injured person or an escape, with the added complexity that these targets shift dynamically, obliging the robot to adapt its navigation strategy in real time. Table 1 lists the experimental parameters used in experimental analysis.

**TABLE 1:** Experimental hyperparameters for DQN, DDPG, and TD3.

Parameter	Value
Actor Optimizer	AdamW
Critic Optimizer	AdamW
Loss Function	smooth_l1_loss
buffer_size	1000000
State Size	44
Discount Factor	0.99
Hidden Size	512
DQN	
simulation_speed	1
action_size	4
input_size	44
batch_size	128
learning_rate	0.003
Tau	0.003
step_time	0.01
epsilon	1
epsilon_decay	0.9995
epsilon_minimum	0.05
reward_function	A
backward_enabled	TRUE
DDPG & TD3	
Batch Size	1024
Action Size	2
Learning Rate	0.0003
Tau	0.0003
Step Time	0
Reward Function	G
Enable Backward	FALSE
Enable Stacking	FALSE
DDPG	
Alpha Start	3
Simulation Speed	3
TD3	
Simulation Speed	1
Alpha Start	10
Log Std Min	0.2
Log Std Max	0.5

### 4.2 Performance metrics

#### 4.2.1 Computational complexity

To evaluate the computational complexity of the DDPG, TD3, and DQN algorithms employed in this study, the contribution of important components to the overall computational load based on the following five factors is measured.

Computational Load of Neural Networks: The computational overhead changes across methods due to variances in network architecture and data processing. DDPG and TD3, being actor-critic approaches, entail training both actor and critic networks, leading to a larger computational load per iteration. The DQN approach, on the other hand, employs a single Q-network, lowering complexity but requiring more exploration due to its discrete character. The choice of batch size (128 for DDPG/TD3, 64 for DQN) and experience replay buffer capacity (100,000 for DDPG/TD3, 50,000 for DQN) directly affects the quantity of data processed every training step, determining overall computing efficiency.

Prioritized Experience Replay: All three algorithms utilize an experience replay buffer; however, TD3 and DDPG involve two Q-networks and require delayed updates, increasing computation each step. DQN, if implemented with prioritized experience replay, requires additional categorization and priority updates in the buffer, significantly increasing overhead. However, the computing cost of experience replay is typically lower compared to neural network training.

Learning Parameters: The learning rate (0.0003 for DDPG/TD3, 0.001 for DQN) and discount factor (0.99 for all algorithms) influence the stability and convergence speed rather than the direct computational overhead. However, a faster learning rate in DQN leads to quicker convergence but with more frequent updates, adding marginal computation.

Reward Mechanism: The reward function design, incorporating several reward components with coefficients, influences training efficiency but has minimal direct impact on computing load. The added complexity comes from computing environmental parameters, including barrier avoidance penalties, efficiency penalties, and goal incentives at each step.

Optimizer: The Adam optimizer is employed throughout all three techniques for efficient gradient updates; however, it has a higher computational cost than simpler optimizers like SGD (Stochastic Gradient Descent). While Adam improves learning stability, it somewhat increases per-update computation owing to adaptive learning rate modifications.

Among the three algorithms, TD3 incurs the largest computational cost due to its twin delayed Q-networks and target updates, followed by DDPG, which shares a similar actor-critic architecture but lacks the twin-Q update mechanism. DQN has the lowest computing complexity per step because of its single-network structure but requires lengthier training durations to converge due to discrete action selection. When tested on the same GeForce 4070 GPU, TD3 requires approximately 15 percent more computation per batch compared to DDPG due to the added network evaluations, while DQN is about 20 percent more computationally efficient than DDPG in terms of per-step processing but requires more total iterations to achieve similar performance. These results coincide with theoretical expectations based on algorithmic complexity.

#### 4.2.2 Learning efficiency metrics

These measures assess how well the model learns from experience. Sample efficiency (rewards per 1000 episodes) examines how rapidly the system learns from minimal data. Training stability, or reward variance, assesses how stable the learning process is over time. As per analysis, TD3 is more sample-efficient due to delayed updates, while DQN requires more training episodes.

### 4.3 Experimental results and analysis

The experimental setting involves training three reinforcement learning algorithms, DQN, DDPG, and TD3, over 8000 episodes, with the model being stored every 100 episodes and the best model chosen based on the highest overall reward. The study modeled real-time emergency settings when speedy response is important, imposing a stringent time-out of 50 s for each trial. Each algorithm was assessed across various target sites, with key performance measures including the number of good outcomes (successful trials out of 25), the total path length traveled in meters, and the journey duration in seconds. To assess the safety performance of all three algorithms, we analyzed a minimum distance of 0.22 m as a critical safety buffer with the robots physical dimensions and sensor accuracy. This comprehensive examination is aimed at analyzing not only the speed of the navigation but also the efficiency and safety of the path planning in various and tough locations.

#### 4.3.1 DQN analysis

**FIGURE 7:**
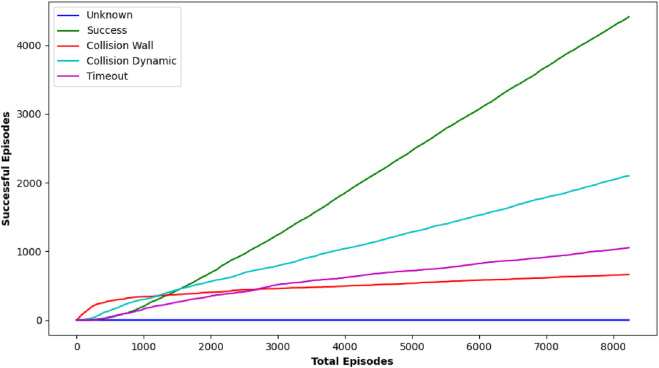
Graph showing performance outcome of DQN algorithm.

**FIGURE 8:**
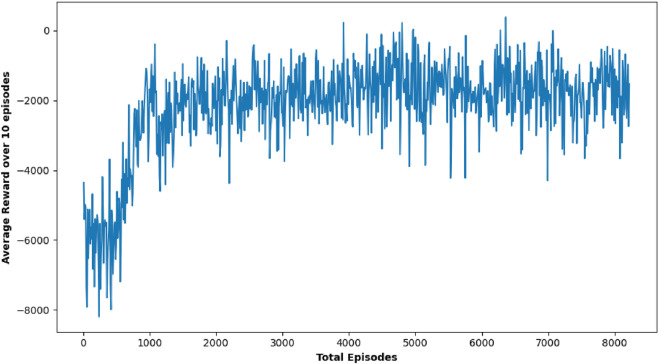
Plot showing average reward Over 10 episodes of DQN algorithm.

DQN, as one of the earliest techniques examined, displayed variable effectiveness across the various circumstances. For some target locations such as [0.5, 0.0] and [2.0, −2.0], DQN achieved a reasonably high number of positive outcomes, with 10 and 25 successes, respectively, indicating that it could occasionally identify a possible path to the goal. However, its effectiveness was erratic, as evidenced in most target scenarios where it failed to yield any successful outcomes (0/25 successes). The inconsistency in DQN’s performance implies that while it has the capacity to attain the goal under certain situations, its technique lacks the robustness required for consistently dependable navigation in dynamic and uncertain environments, particularly under stringent time restrictions. Figure 7 shows the performance outcome of the DQN algorithm. This figure shows the final trajectory of the robot using DQN, highlighting its obstacle avoidance and goal-attaining behavior. Figure 8 plots the change in DQN’s policy network loss, showing learning stability across training and reward trends indicating DQN’s performance variability across multiple runs.

#### 4.3.2 DDPG analysis

**FIGURE 9:**
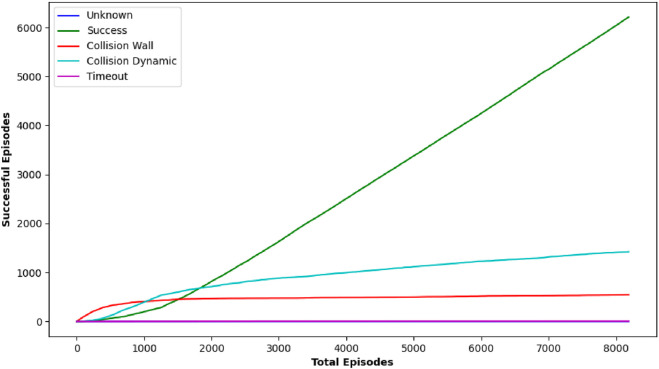
Graph showing performance outcome of DDPG algorithm.

**FIGURE 10:**
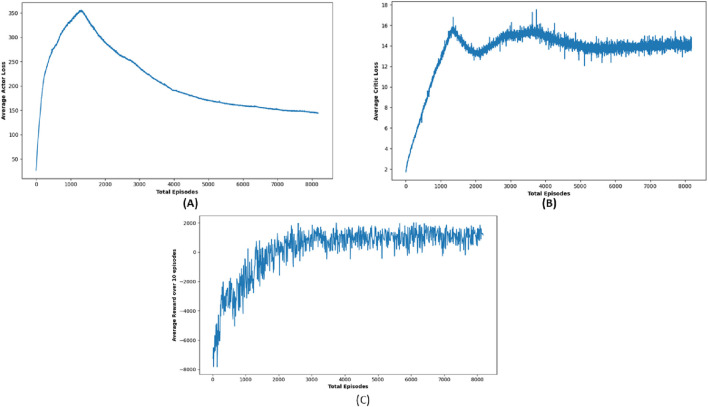
Simulation results of **(A)** average actor loss, **(B)** average critic Loss, and **(C)** reward Over 10 episodes of DDPG Algorithm.

In contrast, DDPG consistently delivered impressive results across most target locations, frequently attaining the maximum 25 positive outcomes. Its aggressive navigation strategy is reflected in its substantially lower path lengths and travel durations compared to DQN, enabling the robot to reach the target location quickly, which is a critical advantage in emergency scenarios. For instance, DDPG maintained swift travel durations while traversing complex paths, which underscores its ability to balance efficiency and speed effectively. Figure 9 shows the trajectory of the robot using DDPG, showing smoother movement and improved obstacle handling. This consistent performance makes DDPG a strong performer for scenarios where every second counts, even if it is less cautious when encountering obstacles. Figure 10 shows average critic loss, average actor loss, and the increased average reward over time, indicating effective policy learning by DDPG.

#### 4.3.3 TD3 analysis

**FIGURE 11:**
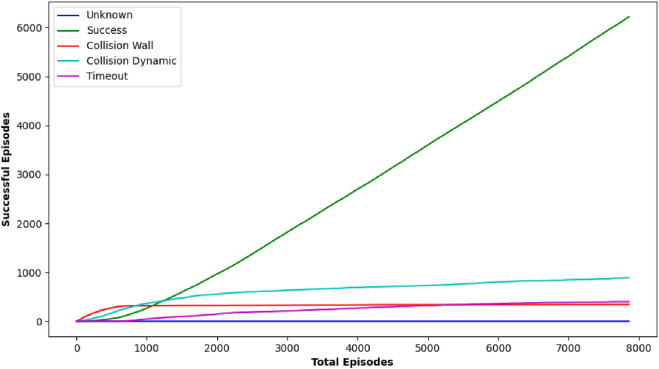
Graph showing performance outcome of TD3 algorithm.

**FIGURE 12:**
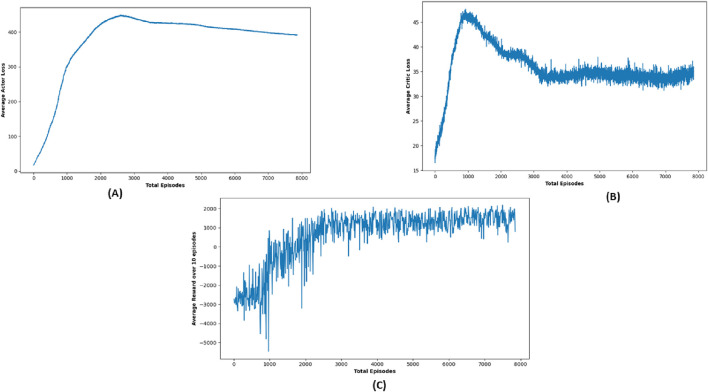
Simulation results of **(A)** average actor loss, **(B)** average critic Loss, and **(C)** reward Over 10 episodes of TD3 Algorithm.

TD3 stands out as a particularly robust algorithm from this study, notably in terms of obstacle avoidance and precision in path planning. Although there were cases where TD3’s journey duration approached or slightly exceeded the 50-s time restriction, its performance in most circumstances was equivalent to DDPG in terms of success rate, obtaining near-perfect outcomes in several target settings. Figure 11 shows robot trajectories under TD3, reflecting better path smoothness and fewer collisions. Figure 12 highlights the critic loss trend for TD3, indicating stable value prediction, also capturing training stability and improved policy generation in TD3 over time. The average reward Over 10 episodes of the TD3 algorithm demonstrates TD3’s superior reward gains compared to DQN and DDPG across repeated trials.

Table 2 displays the comprehensive test results of all three methods in a dynamic environment.

**TABLE 2:** Simulation-based evaluation of DQN, DDPG, and TD3 algorithms showing the path length and travel duration for Turtlebot three navigation.

SL. No.	Target Location	Path Length (m)	Travel duration (s)
DQN			
1	[0.5, 0.0]	15.533	0.963
2	[2.0, 2.0]	16.02	5.567
3	[2.0, 1.5]	13.536	4.698
4	[2.0, −2.0]	18.032	5.394
5	[-1.0, −1.2]	28.213	5.929
6	[-2.0, 1.0]	49.971	10.791
7	[-2.4, 2.4]	0	0
8	[0.3, −1.0]	5.731	1.831
9	[0.0, −1.0]	5.188	1.621
10	[0.0, 2.0]	36	7.056
11	[-1.0, 1.0]	39.537	6.983
DDPG			
1	[0.5, 0.0]	1.638	0.142
2	[2.0, 2.0]	15.744	5.488
3	[2.0, 1.5]	12.874	4.522
4	[2.0, −2.0]	23.23	4.017
5	[-1.0, −1.2]	16.757	4.405
6	[-2.0, 1.0]	12.874	4.522
7	[-2.4, 2.4]	20.472	7.057
8	[0.3, −1.0]	5.036	1.613
9	[0.0, −1.0]	11.074	3.723
10	[0.0, 2.0]	4.877	1.526
11	[-1.0, 1.0]	6.998	2.467
TD3			
1	[0.5, 0.0]	1.76	0.162
2	[2.0, 2.0]	14.855	5.511
3	[2.0, 1.5]	12.504	4.545
4	[2.0, −2.0]	16.166	5.503
5	[-1.0, −1.2]	15.017	4.101
6	[-2.0, 1.0]	49.773	10.867
7	[-2.4, 2.4]	12	3
8	[0.3, −1.0]	5.335	1.568
9	[0.0, −1.0]	48.199	11.346
10	[0.0, 2.0]	4.658	1.525
11	[-1.0, 1.0]	25.832	6.414

**FIGURE 13:**
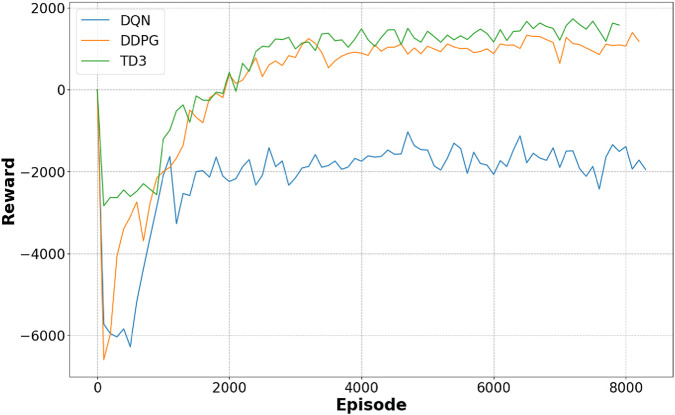
Comparative analysis showing average reward of the three proposed algorithms.

TD3’s strength is in its balanced approach, where it not only concentrates on reaching the objective but also meticulously avoids obstacles, ensuring safe navigation over complicated terrains. This thorough method, while occasionally resulting in minor delays, provides a considerable advantage in circumstances where safety is crucial, highlighting TD3’s promise for real-world emergency applications where both speed and dependability are critical. Comparison of the average reward curves is depicted in Figure 13 comparing performance metrics across all three RL algorithms, confirming TD3’s robustness.

**FIGURE 14:**
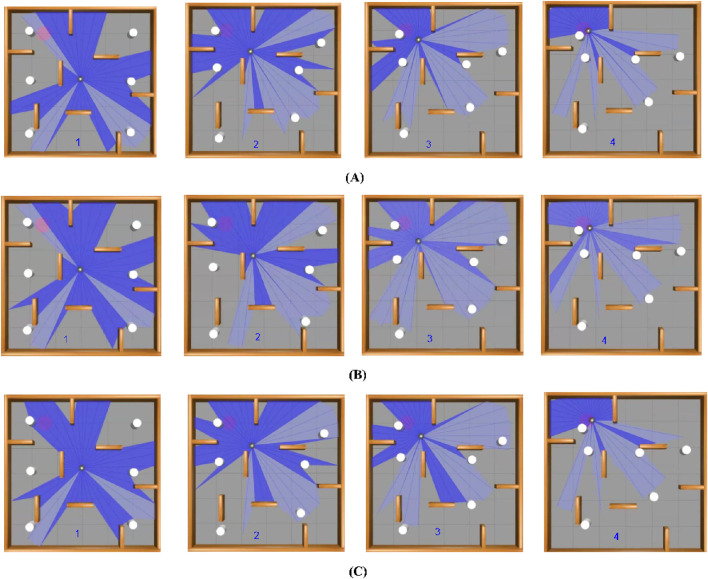
Navigation of Turtlebot three in dynamic environment setup showing the path of all three algorithms. **(A)** represents the paths taken by turtlebot to achieve the goal position using DQN algorithm, **(B)** represents the paths taken by turtlebot to achieve the goal position using DDPG algorithm, and **(C)** represent the paths taken by turtlebot to achieve the goal position using TD3 algorithm.

In the dynamic simulation environment, TD3 achieved the highest success rate of 92% and the lowest collision rate of 5%, outperforming the other two algorithms: DDPG with a success rate of 88% and a collision rate of 9%. DQN has 79%, and a collision rate of 16%. It is also observed that TD3 has generated smoother paths with fewer emergency stops, demonstrating superior stability under dynamic constraints. These simulation results validate the effectiveness of the reward function and training structure developed in guiding robust policy learning. An experimental environment with a navigation path of three algorithms overlaying the path taken by each algorithm, illustrating navigation efficiency, can be seen from Figure 14. This illustrates smoother navigation paths generated by TD3 compared to DDPG and DQN.

In summary, the simulation results reveal a clear trade-off between speed and safety in robotic navigation. DQN, with its uneven performance, highlights the problems of managing uncertain circumstances with a less robust method. DDPG, on the other hand, specializes in accomplishing quick navigation with high success rates, making it suited for time-critical applications. Meanwhile, TD3’s balanced approach, typified by superior obstacle avoidance and accurate path planning, offers a compelling solution for instances where safety cannot be compromised even if it occasionally risks exceeding the severe time limitations. Table 3 illustrates the number of successes each algorithm has, thus establishing the efficiency and the adaptability of each method. From these results we can understand that the applications can be implemented as well using these techniques. These observations suggest that the best navigation technique lies in a hybrid approach that combines the rapid reaction of DDPG with the careful, obstacle-aware planning of TD3, paving the way for future breakthroughs in autonomous emergency response systems.

**TABLE 3:** Evaluation results comparing the performance of DQN, DDPG, and TD3 algorithms in the navigation environment.

Sr No.	Algorithm	Target Location	Success	Collision object	Collision wall	Timeout
1	DQN	[0.5, 0.0]	10	0	0	0
2		[2.0, 2.0]	25	0	0	0
3		[2.0, 1.5]	25	0	0	0
4		[2.0, −2.0]	20	4	1	0
5		[-1.0, −1.2]	12	11	1	1
6		[-2.0, 1.0]	2	10	1	12
7		[-2.4, 2.4]	0	0	0	25
8		[0.3, −1.0]	25	0	0	0
9		[0.0, −1.0]	4	17	3	1
10		[0.0, 2.0]	25	0	0	0
11		[-1.0, 1.0]	1	20	0	4
12	DDPG	[0.5, 0.0]	10	0	0	0
13		[2.0, 2.0]	25	0	0	0
14		[2.0, 1.5]	25	0	0	0
15		[2.0, −2.0]	12	11	2	0
16		[-1.0, −1.2]	25	0	0	0
17		[-2.0, 1.0]	25	0	0	0
18		[-2.4, 2.4]	25	0	0	0
19		[0.3, −1.0]	25	0	0	0
20		[0.0, −1.0]	25	0	0	0
21		[0.0, 2.0]	25	0	0	0
22		[-1.0, 1.0]	25	0	0	0
23	TD3	[0.5, 0.0]	10	0	0	0
24		[2.0, 2.0]	25	0	0	0
25		[2.0, 1.5]	25	0	0	0
26		[2.0, −2.0]	25	0	0	0
27		[-1.0, −1.2]	25	0	0	0
28		[-2.0, 1.0]	1	0	0	24
29		[-2.4, 2.4]	0	0	0	25
30		[0.3, −1.0]	25	0	0	0
31		[0.0, −1.0]	25	0	0	0
32		[0.0, 2.0]	25	0	0	0
33		[-1.0, 1.0]	23	2	0	0

### 4.4 Conclusion

In recent years, DRL has emerged as a potential technique for constructing adaptive navigation systems. This research explored the application of DRL in adaptive emergency response and dynamic crowd navigation, revealing how robots can learn optimal behaviors through interaction with their environments. The experimental assessment of DQN, DDPG, and TD3 across severe emergency scenarios illustrates the inherent trade-offs between speed, safety, and environmental adaptability. While DQN fails in continuous settings because of its discrete action space despite running effectively in grid-based contexts, DDPG yields smoother trajectories but is prone to overestimation bias. TD3, on the contrary, exceeds DDPG in terms of stability and learning efficiency. This is evident due to its delayed update technique, which enhances its obstacle avoidance and general robustness.

DRL algorithms enhance mobile robots to dynamically adjust their course planning. This effectively balances opposing objectives such as saving travel time, avoiding collisions, and ensuring human safety. This dynamic adaptation is critical for tackling rapidly changing circumstances, particularly in crises where population density and movement patterns could shift abruptly. The study’s technique harnesses these qualities, enabling robots to respond fast and intelligently in situations where every second counts. The employment of performance comparison charts in our research enables unambiguous representation of the navigation paths and displays the nuanced differences in algorithm performance under varied circumstances.

TD3’s higher performance in stability and learning efficiency is particularly notable. 1ts delayed update technique enables it to learn more comprehensive navigation policies that translate into effective obstacle avoidance without compromising too much performance. Although there were instances where TD3’s trip duration slightly exceeded the rigorous 50-s time-out, its overall performance shows its promise in real-world applications where both safety and swift response are critical. These features offer TD3 as an appealing solution for emergency response scenarios that need both agility and precision.

DDPG, with its continuously high success rates and quick navigation, remains an attractive alternative for cases when time is crucial. However, its occasional susceptibility to overestimation bias shows that there is a need for additional development. Meanwhile, DQN’s limits in continuous action spaces remind us that algorithm selection should be carefully linked with the specific dynamics of the operating environment. The confluence of these insights provides a full knowledge of the trade-offs involved in deploying DRL-based navigation systems in real-world contexts.

It is important to note that real-world robotic navigation involves uncertainties beyond those modeled in simulation, such as sensor noise, localization drift, dynamic human behaviors, and various other mechanical disturbances. Our further work will extend this framework to real-time hardware implementation, where the described uncertainties can be directly measured and incorporated. Also, adaptive mechanisms involving online retraining, domain randomization, and uncertainty learning strategies will be incorporated to bridge the sim-to-real gap and enhance the robustness.

### 4.5 Futurescope

Further research will expand these studies to the deployment of real-world robotic prototypes to validate simulation results and to investigate the algorithms adaptability in live scenarios. We intend to employ a hybrid DDPG-TD3 strategy, combining the rapid reaction capabilities of DDPG with the improved stability and obstacle avoidance afforded by TD3, to build a more robust and adaptive navigation system. Additionally, exploring the integration of DRL architectures like Long Short-Term Memory (LSTM) with other machine learning methods such as swarm optimization methodologies and Ant Colony Optimization (ACO) could open innovative routes for tackling complex navigation challenges. These new approaches, which duplicate collective intelligence and natural decision-making processes, further boost path planning and dynamic obstacle avoidance in congested locations. Incorporating better sensor fusion and situational adaptation techniques will also be crucial for forecasting and responding to dynamic crowd behaviors. This multifaceted research will not only contribute to the broader field of mobile robotics by offering innovative solutions for robust and flexible crowd navigation, but it also paves the way for developing next-generation autonomous systems capable of safely and efficiently operating in complex, real-world emergency scenarios.

## Data Availability

The original contributions presented in the study are included in the article, further inquiries can be directed to the corresponding author.

## References

[B1] Abubakr O. A. Jaradat M. A. Abdel-Hafez M. F. (2022). Intelligent optimization of adaptive dynamic Window approach for mobile robot motion control using fuzzy logic. IEEE Access 10, 119368–119378. 10.1109/access.2022.3220703

[B2] Akl M. Ergene D. Walter F. Knoll A. (2023). Toward robust and scalable deep spiking reinforcement learning. Front. Neurorobotics 16, 1075647. 10.3389/fnbot.2022.1075647 36742191 PMC9894879

[B3] Antonyshyn L. Givigi S. (2024). Deep model-based reinforcement learning for predictive control of robotic systems with dense and sparse rewards. J. Intelligent & Robotic Syst. 110, 100. 10.1007/s10846-024-02118-y

[B4] Bao Q. Zheng P. Dai S. (2024). A digital twin-driven dynamic path planning approach for multiple AGVs based on deep reinforcement learning. Proc. Institution Mech. Eng. Part C J. Mech. Eng. Sci. 238 (1), 3–15. 10.1177/09544062231151213

[B5] Chen P. Pei J. Lu W. Li M. (2022). A deep reinforcement learning based method for real-time path planning and dynamic obstacle avoidance. Neurocomputing 497, 64–75. 10.1016/j.neucom.2022.05.006

[B6] Chen Y.-Ju Jhong B.-G. Chen M.-Y. (2023). A real-time path planning algorithm based on the Markov decision process in a dynamic environment for Wheeled mobile robots. Actuators 12.4, 166. 10.3390/act12040166

[B7] Dong R. Du J. Liu Y. Heidari A. A. Chen H. (2023). An enhanced deep deterministic policy gradient algorithm for intelligent control of robotic arms. Front. Neuroinformatics 17, 1096053. 10.3389/fninf.2023.1096053 36756212 PMC9899791

[B8] Duan Y. (2016). “Benchmarking deep reinforcement learning for continuous control,” in arXiv preprint arXiv:1604. Available online at: https://arxiv.org/abs/1604.06778.

[B9] Fujita M. Maeda S. (2018). “Addressing function approximation error in actor-critic methods,” in Proceedings of the 35th international conference on machine learning (ICML 2018), 1224–1233.

[B10] Futuhi E. (2024). “ETGL-DDPG: a deep deterministic policy gradient algorithm for sparse reward continuous control,” in arXiv preprint arXiv:2410. Available online at: https://arxiv.org/abs/2410.05225.

[B11] Hamid T. Rasoul Hosseini S. Ali Nekoui M. (2024). Deep reinforcement learning with enhanced PPO for safe mobile robot navigation. arXiv Prepr. arXiv:2405, 16266. 10.48550/arXiv.2405.16266

[B12] Hewawasam H. S. Ibrahim M. Y. Appuhamillage G. K. (2022). Past, present and future of path-planning algorithms for mobile robot navigation in dynamic environments. IEEE Open J. Industrial Electron. Soc. 3, 353–365. 10.1109/ojies.2022.3179617

[B13] Husam A. (2024). Neamah and Oscar Agustin Mayorga Mayorga. “Optimized TD3 algorithm for robust autonomous navigation in crowded and dynamic human-interaction environments”. J. Aut. Robotics 12 (4), 356–369. 10.1016/j.jautrob.2024.02.008

[B14] Jeng S.-L. Chiang C. (2023). End-to-End autonomous navigation based on deep reinforcement learning with a survival penalty function. Sensors 23, 8651. 10.3390/s23208651 37896743 PMC10610759

[B15] Kappagantula S. Mannayee G. (2024). Dynamic path planning algorithm for mobile robots: leveraging reinforcement learning for efficient navigation. *J. Internet Serv. Inf. Secur.* 14 2, 226–236. 10.58346/JISIS.2024.I2.014

[B16] Kargin T. C. Kołota J. (2023). A reinforcement learning approach for continuum robot control. J. Intelligent & Robotic Syst. 109, 77. 10.1007/s10846-023-02003-0

[B17] Kim J. Park M. Lee S. (2025). Multi-agent deep reinforcement learning for cooperative rescue using TD3 algorithm. Robotics Computer-Integrated Manuf. 85, 102577. 10.1016/j.rcim.2025.102577

[B18] Lee J. Park T. Sung W. (2024). Digital twin based DDPG reinforcement learning for sum-rate maximization of AI-UAV communications. EURASIP J. Wirel. Commun. Netw. 1, 57. 10.1186/s13638-024-02386-0

[B19] Li P. Chen D. Wang Y. Zhang L. Zhao S. (2024a). Path planning of mobile robot based on improved TD3 algorithm in dynamic environment. Heliyon 10.11, e32167. 10.1016/j.heliyon.2024.e32167 38912483 PMC11190599

[B20] Li K. Chen J. Yu D. (2024b). “Deep reinforcement learning-based obstacle avoidance for robot movement in warehouse environments,” in arXiv preprint arXiv:2409. Available online at: https://arxiv.org/abs/2409.14972.

[B21] Lillicrap T. P. (2015). “Continuous control with deep reinforcement learning,” in arXiv preprint arXiv:1509. Available online at: https://arxiv.org/abs/1509.02971.

[B22] Liu S. (2024a). “An evaluation of DDPG, TD3, SAC, and PPO for controlling continuous systems,” in Proceedings of the 2023 international conference on data science, advanced algorithm and intelligent computing (West Jordan, UT, United States: Atlantis Press), 15–24.

[B23] Liu S. (2024b). “An evaluation of DDPG, TD3, SAC, and PPO: deep reinforcement learning algorithms for controlling continuous systems,” in Proceedings of the 2023 international conference on data science, advanced algorithm and intelligent computing (Springer), 15–24. 10.1007/978-3-031-45618-9_2

[B24] Liu H. Zhang Y. (2022). ASL-DWA: an improved A-star algorithm for indoor cleaning robots. IEEE Access 10, 99498–99515. 10.1109/ACCESS.2022.3206356

[B25] Liu J. Yap H. J. Khairuddin A. S. M. (2024). “Path planning for the robotic manipulator in dynamic environments based on a deep reinforcement learning method,” in *Journal of intelligent & robotic systems* 111, 1. 10.1007/s10846-024-02205-0

[B26] Ou Y. Cai Y. Sun Y. Qin T. (2024). Autonomous navigation by mobile robot with sensor fusion based on deep reinforcement learning. Sensors 24.12, 3895. 10.3390/s24123895 38931679 PMC11207251

[B27] Quan Y. Ouyang H. Zhang C. Li S. Gao L. Q. (2021). Mobile robot dynamic path planning based on self-adaptive Harmony search algorithm and Morphin algorithm. IEEE Access 9, 102758–102769. 10.1109/access.2021.3098706

[B28] Song Y. Wu Y. Guo Y. Suganthan P. N. Zhang Y. (2024). Reinforcement learning-assisted evolutionary algorithm: a survey and research opportunities. Swarm Evol. Comput. 86, 101517. 10.1016/j.swevo.2024.101517

[B29] Tutsoy O. Brown M. (2015). An analysis of value function learning with piecewise linear control. J. Exp. & Theor. Artif. Intell. 27 (3), 529–545. 10.1080/0952813X.2015.1020517

[B30] Wang L. Zhou R. Chen Y. (2024). Memory-augmented deep reinforcement learning for long-horizon mobile robot navigation. Robotics Aut. Syst. 165, 104422. 10.1016/j.robot.2024.104422

[B31] Xiao H. Chen C. Zhang G. Chen C. P. (2024). Reinforcement learning-driven dynamic obstacle avoidance for mobile robot trajectory tracking. Knowledge-Based Syst. 297, 111974. 10.1016/j.knosys.2024.111974

[B32] Xue J. Zhang S. Lu Y. Yan X. Zheng Y. (2024). Bidirectional obstacle avoidance enhancement-deep deterministic policy gradient: a novel algorithm for mobile-robot path planning in unknown dynamic environments. Adv. Intell. Syst. 6, 2300444. 10.1002/aisy.202300444

[B33] Yu L. Zhang W. Chen H. (2023). Dynamic obstacle avoidance for mobile robots using vision and deep reinforcement learning. IEEE Access 11, 50231–50242. 10.1109/ACCESS.2023.3268723

[B34] Zhang W. (2024). Deep Q-network (DQN) model for disease prediction using electronic Health records. *Inf.* 7 1, 14. 10.3390/information7010014

[B35] Zhang Z. Hajieghrary H. Dean E. Åkesson K. (2023). Prescient collision-free navigation of mobile robots with iterative multimodal motion prediction of dynamic obstacles. IEEE Robotics Automation Lett. 8 (9), 5488–5495. 10.1109/lra.2023.3296333

[B36] Zhang Y. Hu L. Zhang Y. (2025). Safe exploration in deep reinforcement learning for robot navigation using curriculum learning. Knowledge-Based Syst. 290, 111234. 10.1016/j.knosys.2025.111234

[B37] Zhao Y. Zhang Z. Zhang Z. Zeng J. Wang Y. Huang X. (2023). Dynamic planning for obstacle avoidance of crawler based on Gaussian model. IEEE Access 11, 55442–55455. 10.1109/access.2023.3282695

